# Self-selected gait speed - over ground versus self-paced treadmill walking, a solution for a paradox

**DOI:** 10.1186/s12984-015-0002-z

**Published:** 2015-02-21

**Authors:** Meir Plotnik, Tamar Azrad, Moshe Bondi, Yotam Bahat, Yoav Gimmon, Gabriel Zeilig, Rivka Inzelberg, Itzhak Siev-Ner

**Affiliations:** Center of Advanced Technologies in Rehabilitation, Sheba Medical Center, Tel Hashomer, Israel; Department of Physiology and Pharmacology, Sackler Faculty of Medicine, Tel-Aviv University, Tel Aviv, Israel; Gonda Brain Research Center, Bar Ilan University, Ramat Gan, Israel; Department of Neurological Rehabilitation, Sheba Medical Center, Tel Hashomer, Israel; Department of Physiotherapy, Ben Gurion University of the Negev, Be’er Sheba, Israel; Department of Physical and Rehabilitation Medicine, Sackler Faculty of Medicine, Tel-Aviv University, Tel Aviv, Israel; Department of Neurology, Sheba Medical Center, Tel Hashomer, Israel; Department of Neurology, Sackler Faculty of Medicine, Tel-Aviv University, Tel Aviv, Israel; Department of Orthopedic Rehabilitation, Sheba Medical Center, Tel Hashomer, Israel

**Keywords:** Gait speed, Over ground walking, Self- paced treadmill, Virtual reality, Visual flow

## Abstract

**Background:**

The study of gait at self-selected speed is important. Traditional gait laboratories being relatively limited in space provide insufficient path length, while treadmill (TM) walking compromises natural gait by imposing speed variables. Self-paced (SP) walking can be realized on TM using feedback-controlled belt speed. We compared over ground walking vs. SP TM in two self-selected gait speed experiments: without visual flow, and while subjects were immersed in a virtual reality (VR) environment inducing natural visual flow.

**Methods:**

Young healthy subjects walked 96 meters at self-selected comfortable speed, first over ground and then on the SP TM without (n=15), and with VR visual flow (n=11). Gait speed was compared across conditions for four 10 m long segments (7.5 – 17.5, 30.5 – 40.5, 55.5 – 65.5 and 78.5-88.5 m).

**Results:**

During over ground walking mean (± SD) gait speed was equal for both experimental groups (1.50 ± 0.13 m/s). Without visual flow, gait speed over SP TM was smaller in the first and second epochs as compared to over ground (first: 1.15 ±0.18 vs. second: 1.53 ± 0.13 m/s; p<0.05), and was comparable in the third and fourth (1.45 ± 0.19 vs. 1.49 ± 0.15 m/s; p>0.3). With visual flow, gait speed became comparable to that of over ground performance already in the first epoch (1.43 ± 0.22 m/s; p>0.17). Curve fitting analyses estimated that steady state velocity in SP TM walking is reached after shorter distanced passed with visual flow (24.6 ± 14.7 m) versus without (36.5 ± 18.7 m, not statistically significant; p=0.097). Steady state velocity was estimated to be higher in the presence of visual flow (1.61 ± 0.17 m/s) versus its absence (1.42 ± 1.19 m/s; p<0.05).

**Conclusions:**

The SP TM walking is a reliable method for recording typical self-selected gait speed, provided that sufficient distance is first passed for reaching steady state. Seemingly, in the presence of VR visual flow, steady state of gait speed is reached faster. We propose that the gait research community joins forces to standardize the use of SP TMs, e.g., by unifying protocols or gathering normative data.

## Background

The study of gait in health and disease has been the focus of rapidly growing interest in recent years. Gait laboratories which are based on stationary motion capture cameras array, are relatively limited in terms of path lengths and subsequently enable the sampling of an only limited number of continuous strides. Important walking aspects such as gait variability, which require larger number of consecutive strides, cannot be reliably evaluated in these laboratories. On the other hand, gait analysis systems which are based on mobile wearable sensors e.g., forces sensitive insoles, enable the recording of sufficient number of gait cycles, but fall short of providing spatial information, e.g., joint angles, since it is impractical to cover large spaces with motion capture cameras.

Treadmills (TMs) for gait analysis, require small space (i.e., a room to host the TM), and if these TMs are monitored by a motion capture cameras array, a complete set of gait parameters can be obtained with no duration restrictions for the experiments, i.e. without limitation of the number of gait cycles.

The main concern is the observed discrepancy between gait patterns generated by walking on TMs which dictate constant walking velocities, versus those obtained during functional over ground walking [1]. It was suggested that TM walking is different from over ground walking since the TM belt provides ongoing proprioceptive sensory cueing, and since it lacks normal visual flow; while the subject is practically maintaining his/her place in space [2]. Thus, paradoxically, any chosen fixed gait speed on a TM, including the self-selected comfortable walking speed, is not reflecting accurately over ground walking.

Recently, a solution to the paradox has been proposed suggesting that self-paced (SP) walking can be realized on TM with the use feedback-controlled speed which adjusts the TM speed to the user [3]. Most recent studies have compared comprehensively SP TM walking with fixed speed TM walking; using body markers setups and motion capture systems. The results showed that, basically, gait patterns were comparable between SP and fixed speed TM walking [4,5]. However, to the best of our knowledge, explicit comparison with over ground walking performance was not evaluated.

Self- selected gait speed is an important measure in the evaluation of human locomotion and even serves as a target in new diagnostic methodologies [6,7]. For example, slower gait is observed amongst elderly subjects versus younger ones [8,9] or amongst those with neurological [10-12] or psychiatric disorders [13,14], or mixed medical conditions such as depression and Parkinson’s disease [15].

Several functional tests were established to evaluate the subjectively preferred gait speed by human subjects. One of the most popular tests, extensively used for rehabilitation assessment is the 10 meter walking test (10MWT; [16] review: [17]).

Our objective was to compare the self-selected gait speed as evaluated by the over ground 10MWT, to the self-selected gait speed when the subject walks on a SP TM in two experiments. In SP TM EXPERIMENT A the subjects were not exposed to any visual flow (looking at a fixation point), and SP TM EXPERIMENT B used a large scale virtual reality (VR) system that provides realistic visual flow.

## Methods

### Participants

Young healthy adults volunteered to participate in the study. They were included if they reported that they were healthy and free of any clinically significant co-morbidities likely to affect gait, e.g., acute illness, diabetes mellitus, rheumatic or orthopedic diseases, dementia, depression, history of stroke, significant head trauma or brain surgery in the past. The experimental protocol was approved by the Ethical Committee of Human Studies at the Sheba Medical Center. All subjects provided written informed consent according to the Declaration of Helsinki prior to study entry. A total of 26 subjects were recruited, 15 participated in EXPERIMENT A, and additional 11 in EXPERIMENT B. There was no participation overlap between the experiments.

### Apparatus- Experiment A

A split-belt instrumented TM (R-Mill, ForceLink, The Netherlands) placed in a VR facility (V-Gait, Motek Medical, the Netherlands) was used with the tied belts mode solely. Motion capture system (Vicon, Oxford, UK) covers the space occupied by the TM, thus capturing kinematic data from the walking subject from an array of passive markers attached to the subject’s body. Sampling rate was 120 Hz. The VR capabilities of the V-gait were not used, i.e., no visual scenery was presented.

In this study, TM speed was determined in a self-paced mode, i.e., a built-in controller algorithm regulates belts’ speed. The algorithm, which is described in length elsewhere [4,5] generates a control feedback to the TM motor as a function of the instantaneous position and speed of the subject:

1 $$ X^{{\prime\prime} }=P\varDelta X-D\varDelta X\ast X^{\prime } $$

Where *X"* is the acceleration command to the TM motor, Δ*X* is the difference between the subject’s position and the middle of the TM (anterior – posterior), and *X’* is the subject speed. D and P are coefficients set by the manufacturer.

Briefly, positional data from the pelvis markers in the anterior – posterior axis (‘forward-backward’), is utilized in a functional negative feedback loop, i.e., if the subject is moving forward, belts’ acceleration is increased (i.e., to ‘restore’ the subject’s position backward). In the complementary case where the subject slows down, the backwards shift in markers’ position is detected and a deceleration feedback is generated to the TM motor. Pelvis position is calculated as the averaged position of 2 Hz-filtered pelvic markers input, to reduce the influence of marker occlusion and within-stride pelvic fluctuations. TM speed was updated with 30 Hz, using a 6 kW motor per belt.

### Apparatus- Experiment B

**Figure 1:**
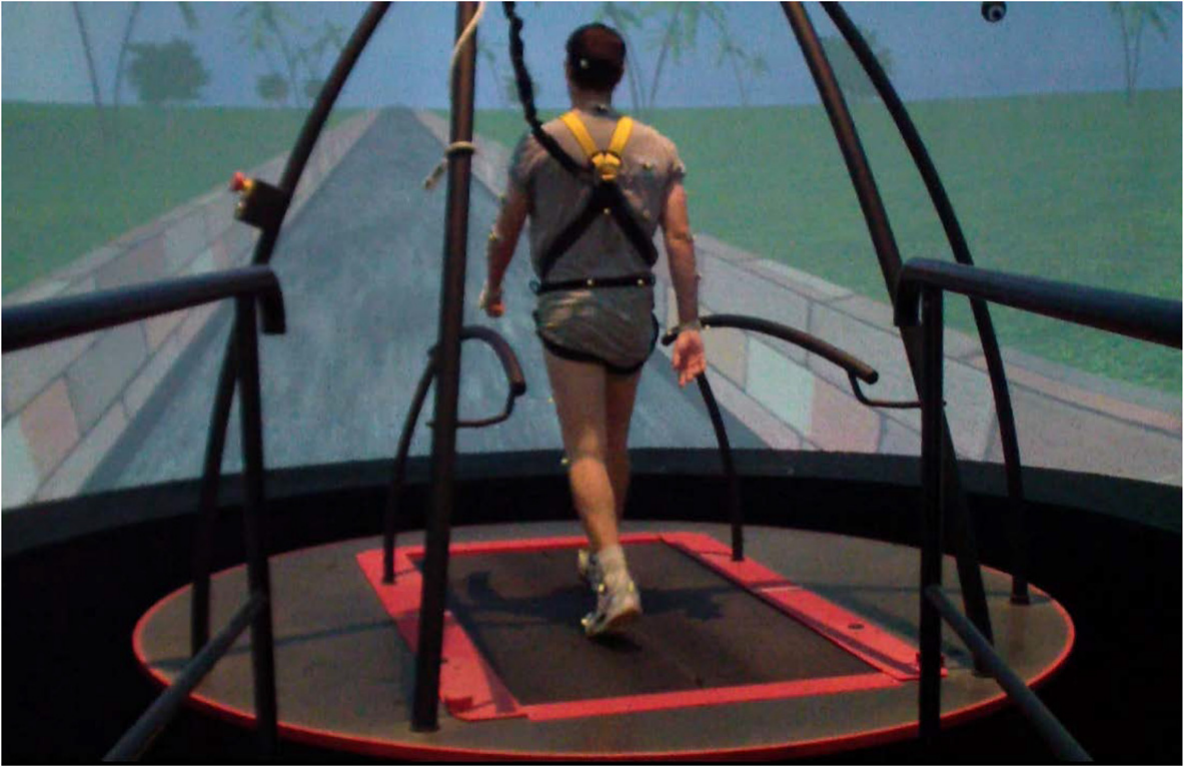
**Experiment in Large scale virtual reality system.** Computerized Assisted Rehabilitation Environment – CAREN High End, consisting of a dual-belt instrumented treadmill (used in tied-belt mode solely) placed in a speed-matched projected visual virtual environment with 360° projection.

A split-belt instrumented TM (R-Mill, ForceLink, The Netherlands) placed in a large virtual reality facility was used (Figure 1, Computerized Assisted Rehabilitation Environment – High End – CAREN High End, Motek Medical, the Netherlands). As detailed in EXPERIMENT A, TM speed was determined in a self-paced mode and identical motion capture system was used.

### Procedure

#### Over ground walking

Each subject performed four consecutive trials of the 10MWT in continuum. There are several variations for performing the 10MWT. The most common in use is when the subjects are instructed to walk at a comfortable, normal pace for 10 m. Only the middle 6 m segment, however, is timed to eliminate the effects of acceleration and deceleration [9,18]. Since our pilot experiments with SP TM walking suggested that many more than 2 meters are needed to stabilize the gait speed by the subjects, (see Results), in the present study, each of the subjects performed four consecutive trials of the 10MWT in continuum.

**Figure 2:**
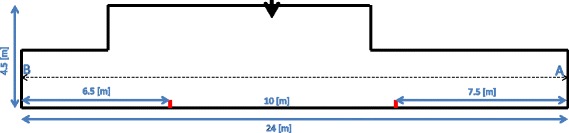
**Scheme describing the path performed by the subjects during over-ground testing.** The subjects walked back and forth in the corridor. The red bars indicate the 10 m marks. These marks were distanced 7.5 and 6.5 m from edges A and B, respectively. Thus, in terms of distance covered, timing of 10 m walking occurred between 7.5 – 17.5 m, 30.5 – 40.5 m, 55.5 – 65.5 m and 78.5- 88.5 m of the overall 96 m walked. Thick black arrow indicates the location of the experimenter.

The subjects were asked to walk continuously back and forth (without stopping) between the edges of a 24 m long corridor (Figure 2; starting from point A walking towards point B) in their own comfortable self-selected pace until they will be asked to stop. The experimenter used a stop watch to time the duration by which a distance of 10 m (pre- marked by small marks on the lower part of the walls) was walked. After returning in the second time to point A, the subjects were informed that the measurement ended.

With these four time values (T), we calculated 4 values of ground speed (GS1, GS2, GS3, GS4; GS_*i*_ = 10/T_*i*[s];_*i* = 1,2,3,4) which were spread over 96 m of walking (see legend of Figure 1 for more details).

### Walking on TM in Self-paced mode

After completing the over ground testing, the subjects were trained to walk on the SP TM on the respective systems for EXPERIMENT A and EXPERIMENT B. This training lasted roughly 10–15 min (including harness restraining, and theoretical explanations), during which the subjects practiced to walk at different speeds according to the experimenter’s instructions. First, they were asked to walk at their preferred comfortable pace. After the subjects reported that they reached their own preferred comfortable walking speed, they were asked to speed up, slow down (even until full stop), and then to retain their preferred comfortable walking speed. This was performed several times until the subjects reported that they feel that they know how to maintain a desired walking speed. During the training minutes for those who participated in EXPERIMENT B, the visual flow was activated (see below).

The SP TM testing trials lasted 100 seconds. In these trials the subjects were instructed to walk in their own self-selected preferred comfortable speed until the system stops.

### Visual flow

In EXPERIMENT A the subjects were presented with a horizontal cross (15 × 15 cm) presented on a monitor about 1.5 meter in front of them and were asked to gaze at it (so that the subjects will refrain from looking at their feet).

Participants in EXPERIMENT B were immersed within virtual visual surroundings. The visual surroundings of the experiment simulate a one-lane road, limited by a short brick fence from either side (Figure 1). When the subject starts walking, the road and the visual surrounding proceed according to the subject’s speed, simulating a ‘real’ walking outdoors.

### Outcome measures

In this study we quantified gait speed. For the over ground walking we used the four time values (T) recorded during the back and forth corridor walking (see *procedure*) in order to calculate 4 values of ground speed (GS1, GS2, GS3, GS4; GS_*i*_ = 10/T_*i*[s];_*i* = 1,2,3,4) which were spread over 96 m of walking (see legend of Figure 2 for more details).

Gait speed during the SP TM experiments was obtained in a post- hoc analysis using computer records from the TMs motor tachometer (sampling rate 120 Hz). Using MATLAB (the Mathwork software) we calculated for each subject, the mean TM speed for 1 meter intervals. In correspondence with GS1, GS2, GS3 and GS4, we calculated the gait speed values for the corresponding TM distances (7.5 – 17.5 m, 30.5 – 40.5 m, 55.5 – 65.5 m and 78.5- 88.5 m – see Figure 2) denoted as TM speed (TMS), i.e., TMS1, TMS2, TMS3 and TMS4 respectively TMi values were obtained by averaging the 10 TM speed values in the respective segments (e.g., TMS1 = mean TM speed over 7.5 – 17.5 m).

### Data handling and statistical analysis

Descriptive statistics are reported as mean ± SD (standard deviation).

Statistical analyses: comparison between gait speeds obtained in different gait segments during self- paced treadmill walking.

We used a two-way Repeated Measures Analysis of Variance (ANOVA) to compare the gait speed values observed during over ground walking with those observed in the corresponding segments during self-paced TM walking. The first factor is the walking condition which has two levels (TM, over ground). The second level is the segment number (4 levels, GS1-4, and TMS1-4, for the over ground and TM walking, respectively). This model was employed separately for EXPERIMENT A and EXPERIMENT B. α = 0.05 was assigned as the level of statistical significance. Additional statistical procedures that were used are detailed in the results section.

### Curve fitting

We employed curve fitting analysis in order to study the effect of the visual flow on the profile of the rise of the gait velocity during self- paced treadmill walking and on gait speed variability during the presumed steady state. For each subject, we established two data vectors, Y, X, where Y is the averaged velocity for every meter walked and X is the accumulated distance (including 0,0 arbitrarily inserted point, and up to maximal distance of 100 meters). These vectors were fitted to the formula:

2 $$ Y=\boldsymbol{a}\mathit{\hbox{-} }{e}^{-bX} $$

Where *a* [m/s] is an estimation of the presumed steady state velocity value, and *b* [m^−1^] provides an estimation of the curvature of the downward concaved function.

To evaluate the level of variability in the gait speed regulation once the subject reaches a presumed steady state level, we first identified the distance passed to the point in which the fitted function crossed the lower value of the 95% confidence interval of the estimated value of *a*, denoted by D_95_ [m]. We then calculated the meter to meter gait speed variability (GS-CV) using the following formula:

3 $$ \mathrm{G}\mathrm{S}\hbox{-} \mathrm{C}\mathrm{V}\left[\%\right]=100\ast \mathrm{C}\mathrm{V}\left(\mathrm{mean}\kern0.5em \mathrm{gait}\kern0.5em \mathrm{speed}\kern0.5em \mathrm{between}\kern0.5em {\mathrm{D}}_{95}\kern0.5em \mathrm{and}\kern0.5em 100\kern0.5em \mathrm{m}\right) $$

## Results

### Participants

Table 1 summarizes the demographic and physical characteristics of the participants in both experiments. It can be seen that cohort composition is similar for both experiments.

**Table 1:** **Demographic and physical characteristics of subjects**

	**EXPERIMENT A**		**EXPERIMENT B**	
**Characteristic**	**Mean ± SD**	**Range**	**Mean ± SD**	**Range**
Age (yrs)	32.57 ± 5.53	24-40.25	29.82 ± 4.31	25-37
Gender (M/F)	7/8		4/7	
Height women (m)	1.61 ± 0.05	1.52-1.68	1.62 ± 0.067	1.54-1.72
Height men(m)	1.76 ± 0.05	1.68-1.8	1.79 ± 0.025	1.76-1.82
Weight women(kg)	56.81 ± 10.16	44-70	59.23 ± 10.05	49-78
Weight men (kg)	77.67 ± 9.59	65-88	79.40 ± 6.52	70-84
BMI	23.32 ± 3.1	17.72-27.43	23.56 ± 3.55	18.34-30.47

*BMI* Body mass index.

### Comparison between over ground gait speed and gait speed on SP TM without visual virtual environment

**Figure 3:**
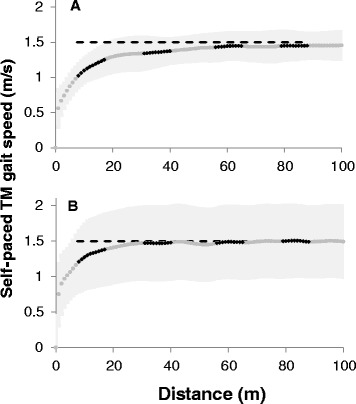
**Self- selected gait speed as function of the distance covered while walking on SP TM.** Gait speed was averaged over each meter walked for each subject, and group grand average is plotted (gray dots). The SD (±) of this average is represented by the shaded light gray area on both sides of the curve. The black short overlapping curves highlight gait speed values during the 10 m segments (TMS1, TMS2, TMS3, TMS4) which are analogous to four consecutive tests of the over ground 10MWT. Dashed horizontal lines in each panel represent the mean over-ground gait speed, 1.50 ± 0.13 m/s. **A.** Data from EXPERIMENT A – self- paced treadmill without the presence of the visual virtual reality scenery. **B.** Data from EXPERIMENT B – self- paced treadmill with the presence of the visual virtual reality scenery. While post hoc comparisons (Bonferroni corrected) yielded no significant difference between TMS3 and TMS4 and GS, mean values of TMS3 and TMS4 showed tendency to be as compared to GS (uncorrected paired *t*-test; p = 0.058, p = 0.038, respectively). TM- treadmill; VR- virtual reality.

Figure 3A depicts, meter by meter, the development of gait speed during SP TM walking, in EXPERIMENT A (panel A). Each data point is the averaged belt speed for one meter of distance passed calculated from the TM tachometer data (light gray data points). The gait speed values during the four designated gait segments are marked by black markers.

The mean values of gait speed (± SD), i.e., TMS1, TMS2, TMS3 and TMS4, were 1.15 ± 0.18 m/s, 1.36 ± 0.20 m/s, 1.44 ± 0.19 m/s and 1.45 ± 0.19 m/s, respectively. The mean OGS value for these subjects was 1.50 ± 0.13 m/s (1.53 ± 0.13 m/s, 1.48 ± 0.14 m/s, 1.49 ± 0.15 m/s, 1.49 ± 0.15 m/s for GS1, GS2, GS3, GS4, respectively – see legend of Figure 3).

The 2 way repeated measures ANOVA model showed a significant *condition* effect (F_[1,14]_ = 14.18; p = 0.002) as well as a significant *segment number* effect (F_[3,42]_ = 28.10; p < 0.001). The interaction condition*segment was found statistically significant (F_[3,42]_ = 28.10; p < 0.001).

Post hoc comparisons (paired *t*-test) showed that TMS1 was statistically significant lower than GS1, and so was TMS2 as compared to GS2 (p < 0.05; α corrected for multiple comparisons). The TMS3-GS3 and TMS4-GS4 comparison yielded no statistically significant difference (p > 0.3).

These results are depicted in Figure 3A. The horizontal dashed line represents the mean values of the OGS measured from the same subjects. A considerable gap between gait speeds measured on the treadmill during the first and second segments and the dashed line is observed, while the third and the fourth segments are tangential to the dashed line.

### Comparison between over ground gait speed and gait speed on SP TM with visual virtual environment

Figure 3B depicts, meter by meter, the development of gait speed during SP TM walking, in EXPERIMENT B. The light gray and black data points represent the averaged belt speed for one meter of distance, and the gait speed values during the four gait segments, respectively.

The mean values of gait speed (± SD) for TMS1, TMS2, TMS3 and TMS4 were 1.43 ± 0.22 m/s, 1.61 ± 0.22 m/s, 1.62 ± 0.18 m/s and 1.64 ± 0.15 m/s, respectively. The mean OGS values for these subjects were 1.50 ± 0.15 m/s (1.50 ± 0.16 m/s, 1.48 ± 0.14 m/s, 1.49 ± 0.18 m/s, 1.50 ± 0.15 m/s for GS1, GS2, GS3, GS4, respectively i.e., almost identical to the OGS obtained in EXPERIMENT A).

The 2 way repeated measures ANOVA model showed a significant *segment number* effect (F_[3,30]_ = 8.30; p < 0.001), but no significant *condition* effect (F_[_1_,_^10^_]_ = 2.42; p = 0.151). The interaction condition*segment was found statistically significant (F_[3,30]_ = 6.67; p = 0.001).

Post hoc comparisons showed no statistically significant gait speed differences between the pairs of over ground gait segments (GS1-4) and the corresponding TM segments (TMS1-4; p > 0.17).

These results are depicted in Figure 3B. While gait speed during the first segment of TM walking was lower than the OGS value (dashed line), the gap was not as considerable as in the EXPERIMENT A. It can also be noted that TMS3 and TMS4 values are slightly higher than GS, while in EXPERIMENT A they were slightly lower.

### Differential effects of self- paced treadmill walking in the presence of virtual visual stimuli

To study the differential effect of SP TM walking in the presence of visual VR stimuli (EXPERIMENT B) as compared to only SP TM walking (EXPERIMENT A), we first normalized gait speed measured in TMS1, TMS2, TMS3 and TMS4 for each subject with respect to the measured OGS and compared the normalized values between the groups (recall OGS values in EXPERIMENT A and EXPERIMENT B were almost identical).

**Figure 4:**
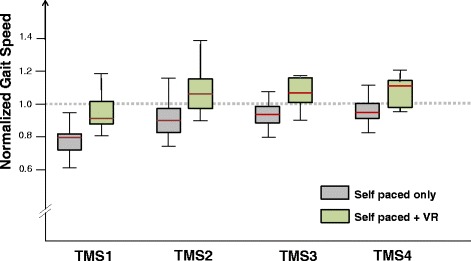
**Differential effect of visual virtual scenery on self- selected gait speed.** For each subject, gait speed values during TM walking were normalized with respect to the GS value. Box plots for normalized TMS1 – TMS4 are plotted for EXPERIMENT A (gray boxes) and for EXPERIMENT B (light green boxes). The horizontal dashed line represents the GS (i.e., = 1).

Multiple non parametric statistical comparisons (Mann Whitney *U*-test) were used to detect a differential effect (Figure 4). It can be seen that generally the relative values of TMS in each of the gait segments are greater in EXPERIMENT B as compared to EXPERIMENT A (p < 0.04; for TMS1, TMS2 and TMS4; p = 0.06 for TMS3; Bonferroni corrected, k = 4).

Comparisons (t-tests; Bonferroni corrected, k = 4) between gait speed values in EXPERIMENT B and EXPERIMENT A (detailed in the paragraphs above) showed significant differences for TMS1 and TMS2 (p < 0.022), and p values between 0.05 and 0.1 for TMS3 (p = 0.095) and for TMS4 (p = 0.055).

### Reaching steady state gait velocity during self- paced treadmill walking

**Figure 5:**
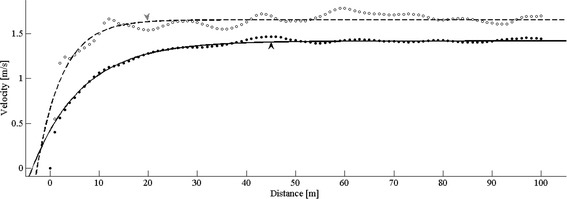
**Estimation of reaching steady state gait velocity values.** Two typical examples of SP TM trials: one without the presence of visual virtual flow (EXPERIMENT A - black dots), and one in the presence of virtual visual flow (EXPERIMENT B gray dots). The respective exponential functions are depicted (solid - and dashed - lines, respectively). In these examples the estimated steady state is higher for the example from EXPERIMENT B (1.66 m/s) and lower for the example from EXPERIMENT A (1.42 m/s), with twice as higher curvature estimation for the former (0.19 vs. 0.09 1/m). It can be seen that in agreement with these estimations, the D_95_ value is about half in the example from EXPERIMENT B (19.9 m gray arrow head) as compared to the example from EXPERIMENT A (45.1 m). Refer to Table 2, for group values and statistical comparisons.

Figure 5 depicts two representative data sets, from SP TM trials, with and without VR visual flow (EXPERIMENT B and A, respectively). It can be seen that the exponential function (c.f., Methods) fits well the data, resembling the shape of the relation depicted for the grand averages shown in Figure 3.

Table 2 depicts the estimated values of the coefficients describing the exponential relation between the development of the gait speed and the distance passed, the estimated distance passed until the subject reached presumed steady state walking (D_95_) and the variability in the regulation of gait speed during the presumed steady state (GS-CV). The regression coefficient values of the fitted function are relatively high, slightly higher for data from EXPERIMENT A (0.94 ± 0.04) as compared to data from EXPERIMENT B (0.90 ± 0.04; p < 0.05).

**Table 2:** **Curve fitting estimated values**

	**EXPERIMENT A**		**EXPERIMENT B**	
**Value**	**Mean ± SD**	**Range**	**Mean ± SD**	**Range**
*a* - Stead state velocity (m/s)	1.42 ± 1.19*	1.15 - 1.79	1.61 ± 0.17	1.37 - 1.86
*b* – Curvature (1/m)	0.15 ± 0.12	0.05 - 0.54	0.18 ± 0.09	0.07 - 0.34
Fit regression coefficient R	0.94 ± 0.04*	0.82 - 0.97	0.90 ± 0.04	0.85 - 0.98
D_95_ (m)	36.5 ± 18.7†	8.2 - 72.2	24.6 ± 14.7	1.8 - 50.4
GS-CV (%)	3.4 ± 1.8	1.1 - 6.6	4.3 ± 1.5	2.0 - 7.1

* p < 0.05; † p = 0.097 (not statistically significant difference;); Mann Whitney *U* Test; one outlier of the estimated curvature *b*, 2.207, was omitted (EXPERIMENT B).

Consistent with the results described above for TMS3 and TMS4 (i.e., Figure 3), the estimated gait speed reached during the presumed steady state phase, is higher (1.61 ± 0.17 m/s) in the presence of virtual visual flow (EXPERIMENT B) as compared to SP TM walking without virtual visual flow (1.42 ± 1.19 m/s; EXPERIMENT A; p < 0.05). According to the theoretical values it is estimated that steady state is reached earlier in the presence of visual flow (~25 m, EXPERIMENT B) as compared to its absence (~37 m, EXPERIMENT A; not statistically significant difference; p = 0.097).

## Discussion

### Summary of findings

The main findings of the present study are:

1. Self- selected comfortable walking speed on self-paced treadmill is comparable to self-selected over ground walking after about 50–60 meters of walking in the case that no VR visual flow is presented (EXPERIMENT A).
2. There is a differential effect when walking on SP TM in the presence of VR visual flow as compared to merely walking on SP TM: (*i*) the subjects already reach a comparable gait speed to their over ground performance, even during the simulated first gait segment (i.e., 7.5- 17.5 m) in the presence of VR; (*ii*) The relative gait speed values (i.e., relative to over ground walking) during the rising phase, and in the steady state phase are higher in the presence of VR (Figures 3, 4; Table 2).
3. It is estimated that steady state walking (in terms of gait speed) is achieved on SP TM, after walking about 24 meters in the presence or about 37 meters, in the absence of VR visual flow, respectively (Table 2).
4. Gait speed values reached in the consecutive sampled gait segments (TMS1- TMS4), are larger when visual virtual reality is present (Figure 4).

## Interpretation of findings

These findings support that SP TMs are a reliable tool to evaluate gait speed. Yet, the findings also underline that walking on a SP TM is not fully identical to over ground walking in terms of gait speed development mostly because the required walking distance to reach a steady state level of gait speed is longer when using SP TM. During over ground walking, 2–3 strides, i.e., ~2 meters are needed to reach a steady constant gait speed [16], whereas in SP TM few dozens of meters are required. This finding is of importance since it shows that the use of SP TM for research of SP walking is reliable within the above-mentioned limitation of distance.

### The role of visual flow in self-paced TM walking

In the present study, the differential effect of the visual flow while walking on SP TM was demonstrated: 1) It is estimated that steady state walking is achieved earlier when visual flow is present; 2) Seemingly, higher values of gait speed and higher acceleration values were seen in the presence of visual flow. Effects of virtual visual scenery on gait were mainly studied with TMs in which the speed was fixed (e.g., [19,20]). We compared our results to studies which used self-paced walking (e.g., [5,21,22]. Observably, the direction of the flow and its expansion rate influence walking speed. Backward moving pattern of the visual flow (i.e., a visual flow pattern corresponding to forward locomotion) reduced free walking speed [23-25] while a forward moving pattern of the flow (i.e., a flow pattern corresponding to backward locomotion), either did not change free walking speed [24] or increased it [23,25]. The heterogeneity of the visual flow methodologies used in these studies (e.g. moving hallway, projection on the floor) does not enable the comparison of their results to ours' which were obtained in an advanced VR setting.

It may be argued, however, that the observation of faster preferred walking speed in the presence of visual flow (as compared to its absence) is in disagreement with the findings of Mohler et al. [22] who reported that higher rates of visual flow in a VR environment elicited significantly lower preferred walking speeds, and also with the findings of Lamontagne et al., [21] who reported a negative linear slope between visual flow rate and preferred gait speed while waking in VR induced by head hamlet.

We propose that the latter findings [21,22] and ours’ are not mutually exclusive. While others described the effect of visual flow rate manipulation on gait speed, we compared gait with or without visual flow where the flow rate was natural and generated by the subject’s walking pace. We suggest that the very presence of simulated natural visual flow facilitates engagement into walking on the TM, as compared to the no visual flow condition, where no visual feedback on rate is provided in response to walking. Therefore it may be concluded that gait speed control, in the presence of visual flow simulated by VR, shows greater similarity to that generated by natural over ground walking. A plausible explanation for this effect is that gait speed control is based on a perception of speed of self-motion that arises from a combination of body-based and visual sensing.

### Practical implications

The present findings together with recent studies that compared SP TM gait performance to that obtained with fixed speed TM [4,5] underscore the benefits of using SP TM:

1. Ability to acquire full spatial- temporal data on gait with no limitations on the ‘distance’ walked, in well simulated over ground walking;
2. Ability to acquire relatively longer terms effects (with time duration depending on the subject’s fitness) of experimental conditions, such as visual diversions, additional cognitive engagement while walking (so called ‘dual tasking’). Studying gait with SP TM is a more natural introduction of circumstantial change during one (or several) continuous walking trial (s) as opposed to separate short (minute or two) trials that allow only a certain degree of mental preparation to the change of circumstances.
3. Presently, it is common to establish the so-called preferred walking speed during short over ground trial, prior to the start of any gait experiment. However, when being tested in the gait analyses laboratories, subjects do not always perform the same ‘self-selected’ walking speed as the one sampled during the over ground walking. E.g., when choosing the speed of a treadmill with a dictated gait speed control the subjects often choose different speed. With the use of SP TM it will be no longer needed to establish the over ground walking speed, since all the experimental conditions can be presented in the same set up. The potential long recording times of spontaneous natural walking, will also allow obtaining temporal gait variability outcomes such as stride to stride time variability, swing time asymmetry and indices of bilateral coordination of gait [26].

Technological advancements are rapidly entering the field of rehabilitation. Among these are different types of treadmills, and the use of VR systems by which subjects are exposed to multimodal stimulations [27]. The present study confirms that after short acclimatization to walking in self-paced mode, i.e., few minutes, gait performance is similar to over ground walking in terms of self-selected gait speed on SP TM,. On the other hand, in most over ground gait speed evaluations, no acclimatization is needed and the subjects are practically tested instantly.

### Limitations

In this study we only focused on gait speed. However, one of the main concerns when assessing gait on TMs, is related to gait variability measures. Hence, further studies comparing gait variability between over ground walking and SP TMs are mandatory.

There are several factors that were not controlled for in the present study. For example, the visual scenery in EXPERIMENT B was not identical to the corridor scenery, in which, the subjects performed the four consecutive 10MWT trials. Similarly, we did not introduce virtual visual scenery using a relatively small flat screen. Instead, the CAREN high end system (Figure 1) that provides high degree of immersion, a relatively rare system, was used. Further exploration of the role of visual flow in particular with respect to its ability to facilitate rehabilitation procedures is required.

These limitations are inherent to the fact that the SP TMs are usually a part of modern systems that have many control variables. The number of degrees of freedom for designing experimental protocols that utilize the modern technologies, such as advanced instrumented TMs and VR systems, is very high. This raises the necessity of a certain of coordinative effort among researchers using these types of systems, in order to be able to standardize, compare, and reproduce scientific results obtained from different research groups.

Thus, at first stage, we propose to establish a common data set of normative data of gait speed values obtained from SP TM, and to establish a standardized testing protocol that will include SP TM parameters, duration of testing and more. For example, it remains to be determined whether the distance needed to reach constant gait speed SP TM is age-dependent and whether age-related norms can be established.

## Conclusions

Self-selected gait speed generated when walking on SP TM is comparable in magnitude to the gait speed values generated during over ground walking, but reaching a constant gait velocity takes longer on the SP TM as compared to over ground walking. The presence of virtual visual flow facilitates the process. This study supports that SP TMs are advantageous tools to study gait: they present flexible experimental duration, abilities to record full body spatial – temporal gait parameters via motion capture systems, and they mimic realistic walking with the use of added visual flow by VR systems (i.e., more ecologic). Since this methodology is relatively new, it is advisable that the community will join forces for standardizing the use of SP TMs, e.g., by unifying protocols or gathering normative data.

## Abbreviations

10MWT: 10 meter walking test
ANOVA: Analysis of variance
GS: Ground speed
GS-CV: Gait speed variability
OGS: Over ground speed
SP: Self-paced
TM: Treadmill
TM: Treadmill
TMS: Treadmill speed
VR: Virtual reality
